# Local Seeing Measurement for Increasing Astrophysical Observatory Quality Images Using an Autonomous Wireless Sensor Network

**DOI:** 10.3390/s20133792

**Published:** 2020-07-06

**Authors:** Raúl Parada, Sergio Rueda-Teruel, Carlos Monzo

**Affiliations:** 1 Faculty of Computer Science, Multimedia and Telecommunications, Universitat Oberta de Catalunya (UOC), Rambla del Poblenou 156, 08018 Barcelona, Spain; cmonzo@uoc.edu; 2 Ctr. de Estudios de Física del Cosmos de Aragón (CEFCA), Plaza San Juan 1, 44001 Teruel, Spain; srueda@cefca.es

**Keywords:** wireless sensor networks, local seeing, thermistor, Zigbee, astrophysical observatory, image quality

## Abstract

Astrophysical observatories (AOs) are used to acquire high-quality images from the sky. However, AOs are amenable to distortion phenomena such as seeing. In this paper, we consider specifically the local seeing produced from indoor and outdoor temperature variations. Local seeing contributes to the captured image quality, therefore it must be measured. Local seeing has been considered, to the best of our knowledge, in observatories using ad hoc solutions, typically with high cost and complexity. This paper presents the complete development of an autonomous wireless sensor network (WSN) composed of temperature-measuring for real-time local seeing measurement. Therefore, a WSN is deployed using Zigbee as a data communication exchange. As a result, a long continuous-operating system is constructed and tested in a real AO infrastructure. Finally, we calculate a preliminary dome local seeing, from the acquired temperature data, achieving reasonable results.

## 1. Introduction

**Figure 1:**
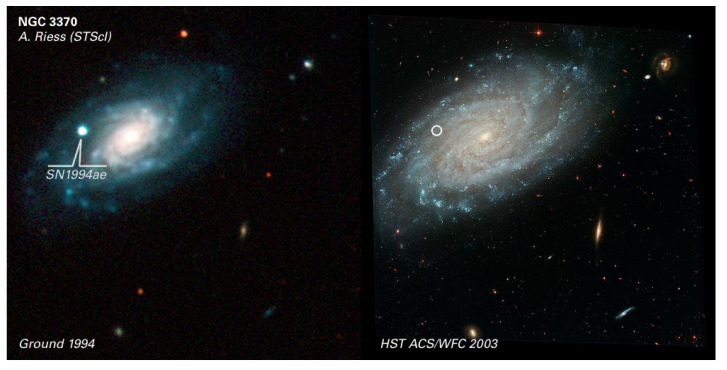
The lower the value of seeing, the clearer the objects from the universe [2].

Astrophysical observatories (AOs) are scientific infrastructures to observe and acquire high-quality images from the sky to compose a complete celestial map, study given phenomenon, and other tasks. Ground-based AOs are placed in high-altitude remote locations to reduce environmental, light, and radio-frequency interference effects. However, AOs are sensitive to distortion phenomena reducing the image quality. One of the most common phenomena is called seeing, roughly categorized into atmospheric seeing and local seeing. Atmospheric seeing depends on atmosphere changes and local seeing it is produced from indoor and outdoor observatory temperature variations, being a more controllable issue to take into account. Therefore, the variations in temperature create turbulence on the dome, which causes astronomical objects to flash and forms blurry images, putting a limit on the capacity of telescopes and instruments to solve astronomical objects. The intuition behind local seeing is that an immobile and optically perfect image indicates an excellent seeing. In opposition, a rapidly changing image generates a distortion and indicates poor seeing. For instance, to get an idea of the seeing’s phenomenon importance, NASA invested 2800 million US dollars in the Hubble Space Telescope to get a 0${}^{\prime \prime }$ seeing [1], as the light that collects does not go through the atmosphere. It is usually expressed in arcseconds; however, they are also used other scales such as Pickering or Antoniadi. It’s values, in places not suitable for observation, easily vary between 2${}^{\prime \prime }$ to 10${}^{\prime \prime }$, while in professional observatories this value improves considerably. For instance, the astrophysical observatory of Javalambre (OAJ) in Teruel, Spain, presents a 0.70${}^{\prime \prime }$ seeing on average. This value is equal than other observatories in Spain such as the Roque de los Muchachos Observatory in La Palma, the Teide Observatory in Tenerife and better than the Calar Alto Observatory in Almería, with a seeing average of 0.90${}^{\prime \prime }$. International observatories include the Paranal Observatory in Chile with a 0.66${}^{\prime \prime }$ seeing on average and the Mauna Kea Observatory in Hawaii 0.43${}^{\prime \prime }$. Note that those values vary slightly according to the year under study. Figure 1 shows how seeing value affects the image quality. The left part corresponds to an image obtained with the 1.2 m telescope at the Fred Lawrence Whipple Observatory. In opposition, the right part images belong to a space image with less seeing than the left one. It is possible to observe how the images from the right part are clearer with respect to the images shown in the left part.

By means of seeing measurement of a site, it is usually performed using a differential image motion monitor (DIMM) [3]. Here, the light is passed off as two identical openings separated by a distance *d*, with the aim of producing two twin images of a star with the same telescope. The cone of light which produces each of these openings is separated using a prism in one of them, and thus forming two separate images in the focal plane of the telescope. The relative movement of both images in the plane focal represents local inclinations of the wavefront. There are factors that will affect equally the images by both openings as the vibrations of the telescope; however, there are other factors that affect each of the openings differently, such as the atmospheric turbulence. As mentioned above, the local seeing is the perturbation contribution that the observatory with its observation elements introduces to the global seeing. The work of D. Blanco [4] identifies the major sources of local seeing and their expressions found in the literature:

- **Indoor’s dome seeing**: It arises from the differences in temperature between the interior face of the dome, the different elements and the temperature of the air inside it. It is calculated following Equation (1) based on the development of the work in [5]:
  (1) $$\epsilon_{IntDome}=0.10\times \Delta T_{D-a}^{1.2}$$
  where $T_{D-a}$ is the difference in temperature between the interior surface of the dome and the temperature of the indoor ambient air.
- **Outdoor’s dome seeing**: Here, the turbulence is originated due to the temperature differences between the outer surface of the dome and the local temperature of the air that surrounds it. It is calculated following Equation (2) based on the analysis performed by the authors of [6,7]:
  (2) $$\epsilon_{ExtDome}=0.12\times \Delta T_{a-s}^{1.2}$$
  where $T_{a-s}$ is the difference in temperature between the outer surface of the dome and the temperature of the ambient air that surrounds it.

In professional observatories, the study of local seeing acquires a great importance, as once it is determined, the corrective measures can be taken for its minimization to achieve the highest possible image quality.

Until a few years ago, the contribution of the thermal difference between the inside and the outside from the observatory was known. Thus, researchers name that contribution as dome seeing. Then, in the mid 1970 s, although its effects were already known, studies on this phenomenon were purely anecdotal, largely because of the difficulty that existed for design and interpreting the results [8]. However, in those years, the commissioner of large telescopes, class 4 m (the term class refers to the size of the primary mirror) and the seeing dome phenomenon begins to draw the attention of astronomers when they realise that the observations made with those large telescopes and enhanced optics, were not able to obtain the expected quality and resolution. With respect to the size of the mirror primary, for instance, the largest optical telescope in Europe, in its category, is in Canarias and has a mirror primary diameter of 10 m [9]. Considering the previous case, the dome seeing was quickly recognized as generated by the turbulence that is created inside it because of air convection. Therefore, the phenomenon itself was simply the subject of conjecture and just a few experiments were performed. Thus, in the successive decades, especially with the proliferation of tests and the increase of data collected, astrophysical researchers begin to study the phenomenon and little by little they begin to understand and improve the facilities to mitigate their effects.

Many relevant examples in the study of local seeing have been performed. Racine et al. published a study [5] in which it was evaluated how the temperature differences between the primary mirror and the air environment and, between the air inside and outside the dome, affected the degradation in image quality. Since 1986, they installed numerous thermistors around the telescope and the dome. Those thermistors were directly connected to a data logger that collected data every 10 min, storing them on a disc. Besides the temperature, they also acquired the speed of the wind. In that way, and using a high-resolution camera, known as HRCam, they could correlate the quality of the images with the temperature differences. Although they already used thermistors to measure the environment temperature, they are wired sensors. Ford, from the Gemini project, published a study [6] of a strategy to seeing control produced by the dome of his installation in Mauna Kea. He created a thermal model of 130 elements to model and simulate the thermal radiation of the sky night, the earth, and the volumetric flow rate of the ambient air from inside the dome. This approach is just a simulation and not a real experiment. Buffa et al. presented a study [10] demonstrating how the temperature gradients among the Galileo telescope mirrors and the air that surrounds them were the primary source of local seeing. They showed how a correct thermalization of the dome during the day, the effect of the local seeing could be mitigated. Specifically, they proposed with a correct weather forecast, the temperature of the air conditioning system from the dome could be fixed. For the weather forecast, they installed a meteorological station at 15 m high tower, locating it at the same height as the primary mirror. The meteorological station was composed of four temperature sensors (standard PT100 with an accuracy of 0.1 ${}^{{}^{\circ}}$C), a humidity sensor, a barometer and a wind speed and direction sensor of ultrasonic type. The system acquired data every 30 s and was sent to the building of the telescope by means of the optical fiber. For the prediction of temperature, a postprocessing of the data was carried out and by using mathematical filters (Kalman filters and neural network procedures) performed the weather forecast. Even though their system seemed complete, it is complex and expensive. Bridgeland and Jenkins presented a work [11] considering mirror seeing within a laboratory. It was a non-intrusive method to use in the telescope as well. Their study included the measurement of temperature variations which provoke turbulence. The temperature measurements used thermocouples (thin wire resistance thermometers) and thermistor micro-pearls (micro bead thermistors) where the variation of temperatures was studied between pairs of sensors. They compared the results by using optical techniques such as a laser helium-neon and an interferometer. They conclude that the variations that were given in the pattern of the interference fringes were consistent with the data obtained by the thermistors and with the turbulence law of the fluids of Kolmogorov. Their approach included expensive optical techniques.

Wilson et al. presented a study [12] about seeing in the William Herschel telescope. They used two instruments: a DIMM type seeing monitor located in the vicinity of the dome that houses the telescope, for the intrinsic seeing of the location and, a seeing monitor based on a Shack-Hartmann wavefront sensor that was installed on the telescope. That last was equipped with a Nasmuth focus that through a third mirror, providing another focal point on one side of the telescope tube, allowed the acquisition of seeing values inside the dome. They were able to compare the measurements taken by both monitors. After the study, they concluded, the contribution to seeing by the dome was not significant. In this case, they do not consider the temperature variation. Sánchez et al. [13] presented a work where they measured the contribution of the surface layer around the observatory from 2.3 m to 15 m of height to the optical seeing. They used a mast with temperature micro-differential sensors, located at seven different heights. Each pair of sensors placed within the same height were separated by a 0.95 m rod. The sensors took samples every 5 ms, and thus, from the difference of temperatures by each pair of sensors, they could measure the refractive index ($C_{n^{2}}$) in the first 15 m. On the other hand, a DIMM monitor was used for 23 nights to determine the intrinsic seeing of the site, determining a 0.84${}^{\prime \prime }$ seeing on average and the average seeing due to the superficial layer of 0.16${}^{\prime \prime }$. They determined that the optical turbulence of this layer has an average contribution of 5.2% of total $C_{n^{2}}$, which corresponds to an average degradation of seeing about 3.2%. They concluded that the presence of trees on the site does not influence, significantly, the seeing due to the layer being superficial.

Blanco presents a work [4] on the estimation of local seeing in the Discovery Channel telescope by the contribution of different elements. He proposed a simple formulation to measure this contribution based on differences in temperature. The study resorted to a thermal simulation software, ThermoAnalytics program, in which you can establish the location of the observatory and the program determines the solar radiation in function of the date, time, etc. Once introduced the model of the dome and other elements and, introducing some estimations such as wind speed at certain points, by finite element analysis, it is possible to obtain reliable results of the thermal evolution of the installation. Thereby, that procedure may predict the thermal variation between different surfaces and, therefore, make a prediction of the evolution of the dome seeing over the entire simulated period. After his studies, he recommended changing the color of the dome from white to silver color. He concluded, the best seeing is achieved in the early hours of the night when the temperatures are the closest to the equilibrium with the air temperature environment. On the other hand, the difference in the wind speed used (4.76 m/s and 2.1 m/s), had hardly any seeing impact of the dome.

Fuensalida et al. presented a work [14] on the correction of the contribution of the dome seeing using the data collected by the g-SCIDAR instrument through the analysis of Fourier. The g-SCIDAR is an instrument that measures atmospheric turbulence profiles by observing binary stars. It provides the location information in height of turbulence layers, movement of these turbulent layers and, therefore, the speeds thereof. As it is observed, a priori, the instrument obtains the vertical structure of the atmospheric turbulence ($C_{n^{2}}$). The technique was based on Fourier analysis to extract from this profile, the contribution of the dome and the mirror seeing. The formulation of this solution is complex where the procedure was based on the observation of binary stars and the use of five consecutive cross-correlations of the frames with scintillation (due to seeing). Thereby, it considers the recognition of the form of the autocorrelation function of the turbulence of the dome in each cross-correlation. Masciadri et al. presented a work [15] on how to characterize the vertical distribution of atmospheric turbulence ($C_{n^{2}}$), all the parameters that are derived from it and the wind profiles for the large binocular telescope. For this, they use the g-SCIDAR instrument during 43 nights. Here, they also study the auto-correlation information from binary stars to create scintillation maps. In these maps, they form three peaks called triplets where the posterior peaks are proportional to the force of the turbulence of the local layers (between 200 m and 250 m and 20 m and 30 m in height), using correlation techniques can estimate the contribution of the leadership. They conclude that the average value of the contribution of seeing from the dome is 0.52 arcsec and that there exists a strong dependence on seasonality.

Potanin presents a work in [16] describing a method, based on a detector from a Shack-Hartmann wavefront sensor, to determine the optical quality of the telescope. And, in [17], Potanin presents a method based on the knowledge acquired in his previous work, for seeing estimation of the dome of the AZT-22 1.5 m. telescopes and 1.0 m. Zeiss telescope Maidanak in Uzbekistan and the ZTSh telescope of the Crimean Observatory in Ukraine, based on the use of a Shack-Hartmann wavefront sensor. The idea behind these types of sensors is that the divergent rays from the stars are passed through a beam divider cube, these duly collimated rays go through a set of lenses that make that each ray focuses on a different charge-coupled devices (CCD) [18] and depending on the variability of these rays can be inferred seeing. Okita et al. presented a work [19] performed on the Antarctic plateau about the measurement of atmospheric seeing and the estimation of the height contribution of the instruments on the level of the snow surface in seeing. They used DIMM in the visible range (472 nm). In this study, it was concluded that the seeing is excellent reaching values below 0.2${}^{\prime \prime }$, obtaining typically seeing values of 0.3${}^{\prime \prime }$ continuously. Moreover, it is determined that the limit height to achieve those values is 11 meters. Guesalaga et al. presented a work [20] to measure seeing based on the spatial-temporal cross-correlation of the samples obtained by five Shack-Hartmann wavefront sensors from the atmospheric turbulence at different altitudes. This method provides useful information for the operation of adaptive optics systems, such as the number of layers of turbulence, their speeds, altitudes, and strength, and a mechanism to estimate the contribution of dome seeing to the global turbulence. Li and Zhang present a study [21] about the importance that environmental changes exert on the performance of telescopes and how, the use of wireless sensor networks for the real-time measurement of such changes, can help the systems of control to take the necessary measures to mitigate its effects.

Domes are mobile mechanisms since they have a non-limited-azimuth movement and it is the slave of the displacement of the telescope (i.e., the slit of the dome should rotate allowing a continuous perfect vision of the telescope without introducing obstacles in the optical path). This movement makes the placement of different measurement devices along the dome surface a complex task because of power feeding, data link connection, and robustness against movements. Furthermore, those measurement devices should not generate heat, as, they would be, sources of turbulence themselves, and therefore, consumption should be limited to the minimum needed. In addition, we indicate that the measurement systems should not emit light, nor any type of electromagnetic radiation that can be picked up by the instruments.

This paper intends to overcome the difficulties for installing a wireless sensor network and innovate for the local seeing measurement. This work presents a solution for seeing determination of the dome D080 (from the OAJ), which is the dome that houses the JAST/T80 telescope, that can be perfectly be extrapolated to the rest of domes, not only of this but for other observatories. We consider the deployment of a distributed wireless sensor network (WSN) to acquire periodic measurements of the temperature at different points. In this way, through the differences of temperature, we can estimate the domes local seeing, which will be contrasted with the determined seeing by the full-width half maximum (FWHM) of the captured images. As a result of our performed work, we present an open source low-cost sensor system to accurately measure both the indoor and outdoor temperatures to calculate the local seeing in real time. The final aim is to adjust the dome configurations to capture high-quality images from the observatory. Specifically, we achieved the following contributions.

- A comparison of thermistor devices to increase temperature reading accuracy and reducing the node power consumption.
- A construction of sensor nodes with data transfer capabilities using the Zigbee technology.
- A deployment of a wireless sensor network to acquire temperature data along the dome.
- A graphical user interface environment to receive in real time the measured temperature.

The remainder of this paper is organized as follows. Section 2 presents the design procedure to develop the sensor collector system. We describe the construction of the sensing system, performing real tests in Section 3. In Section 4, we discuss the product by highlighting the results, insights, and future improvements. Finally, the paper is concluded in Section 5, summarizing our contributions.

## 2. Materials and Methods

This section presents all the experiment set-up, reasoning about the election of key elements in this work.

### 2.1. Experimental Facilities

The OAJ is a Spanish singular scientific and technological infrastructure (ICTS) located in the *Sierra de Javalambre*, in Teruel. Specifically, it is built in the *Pico del Buitre* (40º02${}^{\prime }$30.58${}^{\prime \prime }$ North, 01º00${}^{\prime }$58.58${}^{\prime \prime }$ West), at 1957 meters of altitude, in the town of *Arcos de las Salinas*. The fundamental objective of the observatory is the realization of large sky astronomical surveys. The astrophysical observatory of Javalambre (OAJ) consists mainly of two professional telescopes of a great field of vision, field of view (FoV), with quality of the image in the whole field: the Javalambre Survey Telescope (JST/T250), a telescope of 2.55 m opening, with a field of vision 3 square degrees, and the Javalambre Auxiliary Survey Telescope (JAST/T80), an 80 cm telescope of opening, with a field of vision of 2 square degrees. Both telescopes are equipped with state-of-the-art panoramic cameras whose large format CCDs, together with specially designed optical filters, form a unique set to perform a mapping of the universe. The camera that equips the JST/250 is known as JPCAM and was, in the moment of its manufacture, the largest chamber in the world (today, occupies the second place) with 14 large format CCD detectors of 9200 × 9200 pixels 10 μm. On the other hand, the filter system consists of 54 narrow filters (∼12.5 nm) spaced between 350 nm and 1000 nm and 2 broadband filters for obtaining photometric redshift precision that nobody to improve it, has achieved so far to the best of our knowledge.

In the OAJ, there is currently a set of monitors to measure the quality of the sky, a DIMM monitor of seeing among them. This monitor allows to quantify the total contribution of seeing due to turbulence, however, does not allow discretizing the local contribution that the domes themselves introduce into seeing degradation. Seeing monitor is not located in the same facility as telescopes; therefore, this value is taken as a reference, reducing the optical quality of the image. The degradation in optical quality is reflected in the worsening of the point spread function (PSF) and the width at FWHM of whose values can be deduced the amount of total degradation that affects the image. The procedure for obtaining this deterioration is based on the calculation of the PSF through the FWHM. In astrophysical centres a software called SExtractor [22] is used, which by calculating the distribution of the energy in the images, the number of photons per pixel, and thus, the FWHM is obtained. Once the FWHM obtained and knowing the plate scale value of the CCD, the PSF can be determined. This PSF uniquely is the degradation of the image and that ideally should match the site seeing. Unfortunately, that is not the case due to the distortion also including all the elements involved in the observation (i.e., surface of the dome). Following the previous reasoning, we decided to measure the temperature variations among different points inside the observatory. More specifically, measure the temperature variation between the surface of the dome and the environment that surrounds it. Thereby, this measurement may establish a relationship between this temperature variation and the variation in image quality, and thus, determine the contribution of the dome to the local seeing.

### 2.2. Communication Module

One of our contributions to the field of local seeing measurement is the deployment of a wireless sensor network to ease the exchange of data among nodes. Even though there exists a large variety of wireless technologies to transmit information on industrial environments, we must consider the following factors to choose the most suitable for our objectives.

- **Reliability**: The sensors can fail due to blockages, energy losses, environmental damage, interference, etc. The failure of any sensor node should not affect the overall operation of the network. Therefore, the network should continue operating in the event of operational losses of one of its nodes.
- **Simplicity**: Wired sensors increase both the deployment and the maintenance complexity and, limiting their mobility along the space. Wireless sensors are suitable to simplify the network design by solving the above-wired issues.
- **Scalability**: The number of sensors deployed in the environment to study can range from a few nodes to thousands of them. Sensor networks should allow adding and eliminating nodes according to the needs of the task to be performed.
- **Topology**: The working configuration mode of a network is key to assure a communication flow between all nodes.
- **Size**: The nodes should adapt to any environment for a fast and simple deployment, and thus, the smaller the hardware component, the better.
- **Consumption**: As the nodes in the sensor networks can only be equipped with a limited power source, the maximum time of use of it is essential.

According to the previous list of factors, and taking into account the different available wireless technologies analyzed previously in [23], we decided to choose Zigbee as the communication technology to exchange data between nodes. More specifically, the company Digi [24] provides Zigbee nodes called XBee to deploy personal networks easily. In addition, the modules implied on the nodes allow the possibility to read both analogue and digital inputs, reducing complexity to include additional microcontrollers. A Zigbee XBee kit is available by Digi to work in a mesh configuration. Since this kit allows the increment of nodes, it can be scalable and, considering the mesh configuration, it assures a high reliability. The small size of each node (2.199 × 3.4 × 0.305 cm) allows its installation in most environments). The total consumption along the nodes’ operation can vary by modifying functionality parameters.

**Figure 2:**
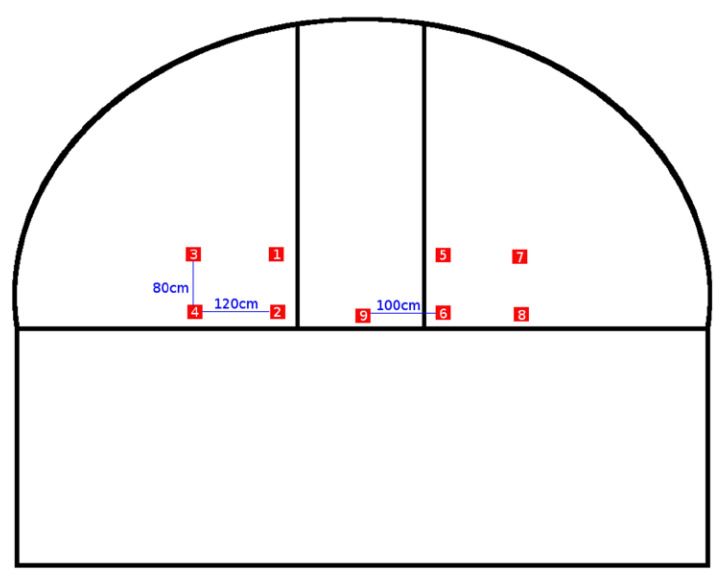
Distribution of the slave nodes along the dome.

Our network is composed of ten nodes where nine of them act as slaves (collect and transmit temperature readings) and one node is the master or collector. The reason for nine nodes to cover the whole dome is because of its dimension. The dome has a diameter of 6.25 m giving a circumference of 19 m, therefore, an approximate resolution of 1 node per every 2 m. With nine nodes, we receive a measurement every 40${}^{{}^{\circ}}$, enough for preliminary experiments. Nevertheless, since these nodes can be easily deployed, we can place them where and when required. Figure 2 shows a scheme of the observatory T80 from an exterior and front view. On the upper part, the dome, it is possible to observe the distribution of the slave nodes to cover the whole surface efficiently.

Note that the distribution of nodes shown in Figure 2 is to measure the turbulence effect at the entrance window from the dome. Nevertheless, we plan other node distributions along the dome.

### 2.3. Temperature Sensors

Temperature sensors are presented as another key element when designing the system since its choice marks the accuracy of the measurements obtained. We discuss our requirements for a suitable sensor in Section 2.3.1. Section 2.3.2, Section 2.3.3 and Section 2.3.4 present the main available temperature sensors for our proposal. We elect the most suitable sensor, with a brief sensor comparison, in Section 2.3.5. Finally, sensor calibration is described in Section 2.3.6.

#### 2.3.1. Preliminary Requirements

As the XBee module admits analogue inputs, it is set as a desirable objective to use analog sensors of low voltage, preferably a maximum 3.3 V. It is desirable in order to take advantage of the power supply from the node, and ideally with output up to 1.2 V to be compatible with the voltage of operation of the inputs of our XBee modules. Otherwise, it would be necessary for the inclusion of another micro-controller, which, among other reasons, that fact would affect the consumption and the management of the nodes in standby mode. The range of temperatures to be measured will be within the range of −20 ${}^{{}^{\circ}}$C to 40 ${}^{{}^{\circ}}$C. We checked previously registered values of the observatory locations, and we also considered a margin of safety of several degrees centigrade. Once the need to use analogue temperature sensors is established, we estimated the minimum necessary precision for an accurate system. According to [4], the seeing variation by turbulence is expected to be in the order of 0.10${}^{\prime \prime }$/${}^{{}^{\circ}}$C ×ΔT to 0.12${}^{\prime \prime }$/${}^{{}^{\circ}}$C ×ΔT, where ΔT is the difference in temperature between two measured points. Note that due to manufacturing reasons, the distance between a pair of sensors from each node cannot be greater than 6 cm, worthy to measure differences between a surface and its upper air layer. However, in case of further distances, it requires the use of two independently nodes separated on the desired distance. The maximum distance between two sensors on the same side of the node is ~4.5 cm, and between nodes of different sides is 9 cm. As each node has two pairs of sensors, two of them are used to measure the dome surface temperature, where there is a direct contact, and the other pair of sensors the distance is about 12 mm. If high precision is desired the position has to be strictly defined, although in our opinion variations of about 1 cm will not be significant, taking into account the results of measurement can be averaged.

Seeing precision required in OAJ is in the order of a tenth of a second of arc and never greater than hundredth of a second of arc. Therefore, it needs the use of sensors with a minimum of precision values. For instance, if a sensor has a precision in the measurement of 2 ${}^{{}^{\circ}}$C, the maximum difference error introduced per pair of sensors would be 4 ${}^{{}^{\circ}}$C, resulting in an estimated maximum error of 0.6${}^{\prime \prime }$, which is an unacceptable value for our installation. Among all the possible temperature sensors available on the market, we selected three models: TMP36 [25], LMT84 [26], and PR103J2 [27]. Those models were selected due to they have analogue inputs avoiding the use of additional controllers and then, increasing complexity. Hence, each analog input has their own analogue-digital (AD) converter easing the connection between sensor and the micro-controller. The XBee AD converter provides a 10 bits resolution, resulting in 1024 possible values. The XBee module allows a range of measurements of 1.2 V, then, the maximum resolution because of quantification is 1.17 mV (1.2 V/1024).

#### 2.3.2. TMP36 Sensor

It is a low-voltage analogue voltage sensor, voltage feeding between 2.7 V and 5.5 V with an accuracy of ±2 ${}^{{}^{\circ}}$C, a typical linearity of 0.5 ${}^{{}^{\circ}}$C, a variation of 10 mV/${}^{{}^{\circ}}$C and a range of measurements ranging from −40 ${}^{{}^{\circ}}$C to 125 ${}^{{}^{\circ}}$C. It suits within the temperature range that we have set. The sensor output voltage is within the range of 1.2 V, admitted by module inputs. With respect to the analogue-digital converter part, this variation of the voltage versus the temperature makes that the maximum resolution is

(3) $$Resolution=\frac{1.17\;\mathrm{mV}}{\frac{10\;\mathrm{mV}}{{}^{{}^{\circ}}C}}=0.117\;{}^{{}^{\circ}}C$$

The TMP36 sensor has been tested with good results; however, its value precision of 2 ${}^{{}^{\circ}}$C, as mentioned in the previous requirements, invalidate completely the measurements obtained so the use of this sensor is underestimated.

#### 2.3.3. LMT84 Sensor

It is another low-voltage analogue temperature sensor. Its typical accuracy is 0.4 ${}^{{}^{\circ}}$C with a variation of −5.5 mV/${}^{{}^{\circ}}$C and its temperature range goes from −40 ${}^{{}^{\circ}}$C up to 150 ${}^{{}^{\circ}}$C. Given this temperature range, the voltage output is from 1.3 V to 0 V, respectively. Apparently, it suits our requirements. Although its precision in temperature is translated into values of one tenth of a second of arc, we could consider it within the established limits. With respect to the AD converter, as we have seen, it quantifies in steps of 1.17 mV, resulting in a resolution of:

(4) $$Resolution=\frac{1.17\;\mathrm{mV}}{\frac{5.5\;\mathrm{mV}}{{}^{{}^{\circ}}C}}=0.3\;{}^{{}^{\circ}}C$$

Considering our requirements, this sensor is within the margins. The resolution of 0.3 ${}^{{}^{\circ}}$C implies a maximum error between two measurements of 0.6 ${}^{{}^{\circ}}$C, resulting in an error of 0.09${}^{\prime \prime }$.

#### 2.3.4. PR103J2 Sensor

In order to increase the precision of the measurements, we studied a high-precision negative temperature coefficient (NTC) thermistor. It is a thermistor of the PR103J2 series from the US Sensor/Littelfuse Inc house. The main characteristic of this sensor is that its resistance tolerance is 0.05 ${}^{{}^{\circ}}$C providing precision values of seeing a hundredth of a second of arc. The temperature range of this thermistor ranges from −55 ${}^{{}^{\circ}}$C to 80 ${}^{{}^{\circ}}$C, which fits perfectly in the ranges that we have established. Note that the resistance of the thermistor is dependent on the ambient temperature. Thus, the manufacturer provides an Excel sheet where a table is displayed with the variation of the resistance as a function of temperature. Therefore, the resistance value for our temperature limits −20 ${}^{{}^{\circ}}$C and 40 ${}^{{}^{\circ}}$C is 97081.38 Ω and 5326.04 Ω, respectively. The default maximum voltage is 3.3 V. As we would like to achieve a maximum value of 1.2 V in the inputs of the module, we use a voltage divider. The voltage divider has been performed by serializing with the thermistor a resistance of 210 kΩ (tolerance ±0.1%). The voltage inputs achieved for our margins are

(5) $$Voltage_{-20{\;}^{{}^{\circ}}C}=\frac{3.3\;V\times 97,081.38\;\Omega }{97,081.38\;\Omega +210,000\;\Omega }=1.04\;V$$

(6) $$Voltage_{40{\;}^{{}^{\circ}}C}=\frac{3.3\;V\times 5326.04\;\Omega }{5326.04\;\Omega +210,000\;\Omega }=0.081\;V$$

That means, there is a variation of 0.96 V in this 60 °C, and therefore a variation of 16 mV/°C. Thus, the resolution of the AD converter gives us a value of

(7) $$Resolution=\frac{1.17\;\mathrm{mV}}{\frac{16\;\mathrm{mV}}{{}^{{}^{\circ}}C}}=0.07{\;}^{{}^{\circ}}C$$

Finally, note that the resistance in series to the thermistor, with a tolerance ±0.1%, results in an error of the same order in the voltage measurements and, consequently, negligible.

#### 2.3.5. Sensor Election

As seen above, both the LMT84 and the PR103J2 sensor, of low tolerance, would fit the requirements. However, there are three factors to consider:

- The highest resolution precision results are achieved with the NTC thermistor.
- The price of the NTC thermistor (including the resistances) is almost ten times higher than the LMT84.
- The consumption is of 50 μA and 5.4 μA for the LMT84 sensor when it is measuring and on standby, respectively. While in the worst-case scenario using the NTC thermistor continuously (with the voltage divider) it is about 15.3 μA (see Equation (8)).
  (8) $$Current=\frac{3.3\;V}{5326.04\;\Omega +210,000\;\Omega }=15.3\;\mu A$$

Table 1 summarizes the characteristics of the tested temperature sensor devices to highlight our election.

We can observe from Table 1 how both the LMT84 and the PR103J2 sensor stand out in one or more characteristics with respect to the TMP36 device. Even though the PR103J2 sensor seems a more complex device, it provides some advantages such as high-voltage range flexibility (1), the scale factor can be easily calculated using the manufacturer curves (2), linear adjustment (3), and the power consumption in our case is between 2.81 μA and 0.015 mA (4).

As a conclusion, all the disadvantages of the NTC thermistor (price and voltage divider) are assumably compared to the great advantage in precision. Therefore, it is decided to use the PR103J2 sensor for data acquisition.

#### 2.3.6. Sensor Calibration

As discussed, the PR103J2 sensor changes its resistance depending on the temperature. An inconvenient of using the thermistor is the non-linearity behavior, resulting in imprecise temperature calculations. Below, we present some possible solutions:

1. Linearize the behavior with a circuit in a trade-off of the required precision measurements.
2. Use the parameter B0/50 from the manufacturer and the Steinhart-Hart equation [28] to convert the values of resistance in temperature. However, the table resistance/temperature from the manufacturer and the results do not match due to the lack of second order parameters.
3. Perform a quadratic regression from the values by the manufacturer to find the most fitting curve. For this purpose, it has been used Matlab, specifically the curve fitting tool, obtaining the equation that fits the data provided and whose root-mean-square error (RMSE) is of the order of the hundredth of degrees Celsius:
  (9) $$Temp^{{}^{\circ}}C=574.1\times Resistance^{-0.1242}-158$$

Another aspect to keep in mind when using thermistors is that, as it is a voltage divider, the voltage value at the input depends on the voltage of supply and that this may vary due to battery depletion. Thus, taking advantage of the fact that this voltage is the same as the power supply to the module, to perform the conversion of the input to resistance, the module performs the measurement of the supply voltage that will be used by the software for calculations. Given that, knowing the input voltage and the supply voltage applying the law of Ohm, it is very easy to know the resistance of the thermistor.

Finally, indicate that the input voltage values have been measured throughout the different tests and no significant differences were found with the results obtained by the software. Voltage differences were of the order of 1 mV; therefore, of the same magnitude as the quantification error of the AD converter, therefore it is considered that these measurements are consistent with the data provided by the manufacturer and that meet the requirements set for this project; however, it does recommend in longer lead times, and with better calibration systems more precise control of the precision in the measurements.

### 2.4. PCB Fabrication

**Figure 3:**
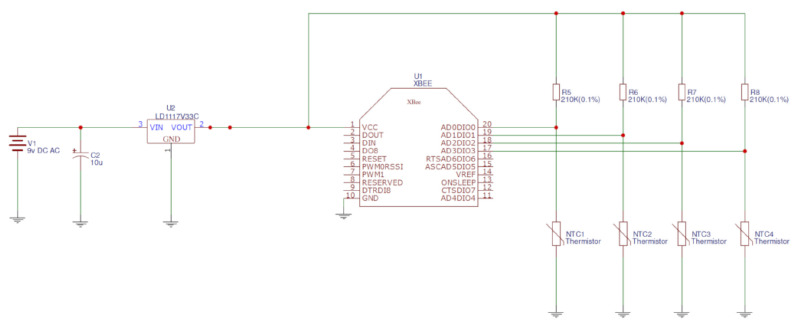
Node electrical diagram.

Once the communication module and the temperature sensor have been selected, we must merge them on an electrical board to use all their capabilities. The first step is the design of the electrical scheme. Figure 3 presents the electrical scheme of the XBee module with four PR103J2 sensors. We decided to use four PR103J2 Thermistor sensors per node to exploit all four analog inputs from the XBee module (named ADCx from 0 to 3) without the need to connect additional controllers. This scheme has been designed using the software EasyEDA v5.1.3.

**Figure 4:**
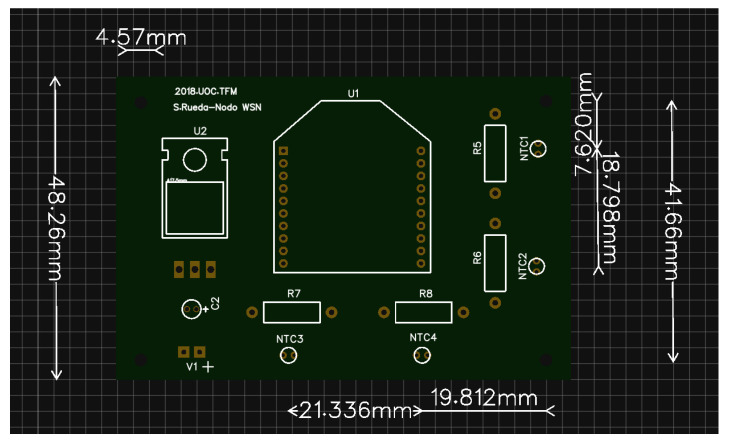
Printed circuit board (PCB) front view from the sensor node using the EasyEDA.

**Figure 5:**
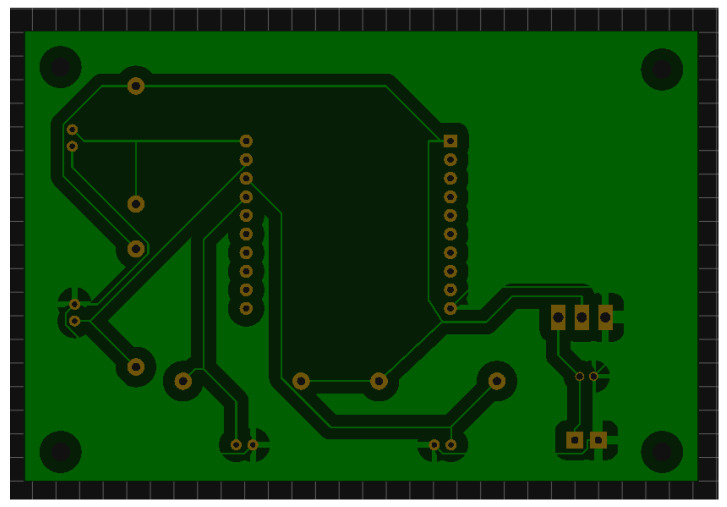
PCB back view from the sensor node using the EasyEDA.

After the completion of the electrical diagram, we draw the printed circuit board (PCB) to lay all the electrical components. Obviously, it requires an energy source to feed the whole system. The election of the battery will be discussed in Section 2.5. The space to allocate the battery has been designed to allow the substitution of different models for testing purposes. Figure 4 and Figure 5 present the front and back design of the PCB, respectively. We designed the PCB to place the following elements.

- Energy source (battery) system:
  - Two pole terminal block, 2.54 mm for PCB
  - 9 V battery connector (4${}^{\prime \prime }$)
  - Micro switch (on/off)
  - Battery element (c.f. Section 2.5)
- Tension conditioner system:
  - Electrolytic capacitor 16V 100 μF
  - Tension regulator LDO 3.3 1 A
- Node and conditioning system for PCB:
  - Digi XBee S2C node
  - 10 pins socket
- Measurement elements:
  - Thermistors PR103J2 10K 0.05 °C
  - Low tolerance resistance 201 KΩ

To manufacture the final PCB version, we sent the circuit files to the professional company JLCPCB [29].

### 2.5. Battery System

In WSNs, the choice of the battery is usually a vital fact, as its duration, in case the energy does not come from an inexhaustible source, will mark the useful life of the sensor or at least the operating time autonomously. The total capacity of a battery is measured in milliampere hours (mAh) or ampere-hours (Ah). For instance, a four ampere-hour battery can supply 4 A for one hour or 1 A for four hour.

When designing the battery system, we must consider, at least, two main aspects [30]:

- The temperature conditions can affect the performance of the batteries. Thus, in environments with non-extreme temperature conditions, the batteries are well preserved. On the contrary, in both very hot and cold environments, batteries may lose effectiveness and not be able to provide the expected performance. The OAJ is a center where temperatures tend to be very mild in summer, so this point would not affect the battery. However, the environment turns extremely cold in winter, and therefore, we should take this point into account. Moreover, future tasks may include carrying out measurements in warmer environments (generator room, electrical panels, etc.), then, the battery performance could also be affected.
- The actual capacity of the batteries is not linear and depends on supplied current. Even though the manufacturer provides us with a nominal load, it is worth bearing in mind that the real one will depend on supplied current.

We should indicate that the XBee modules need a power supply of 2.8 V to 3.4 V and its consumption is of [31]

- 45 mA (maximum) in transmission mode,
- 50 mA (maximum) in reception mode, and
- <10 μA in sleep mode.

Indeed, the election of the battery is key for a low-cost and autonomous wireless sensor node. Among all battery possibilities, we have studied the following because of market availability and electrical characteristics:

- **Alkaline**: It is the most common type of battery for domestic use, its main advantages are its low cost, the fact that they can be easily acquired, and that they are ideal for applications with low current demand at ambient temperature. However, they have two drawbacks: the first is that their performance declines a lot for high current consumption and second and more important in our case, that their performance gets much worse at very cold temperatures, which occurs in winter at OAJ.
- **Lithium**: There is a wide variety of lithium batteries on the market, however, they all have a common behavior, and they work better at low temperatures than alkaline batteries, however, their price is multiplied by a factor of three or four if we compare with these alkaline batteries.
- **Nickel–metal hydride**: It is high quality rechargeable batteries, one of its advantages is that they do not lose performance after many recharge, something that happens with the nickel-cadmium battery. This kind of battery can be considered as an improvement of the alkaline since they behave better at low temperatures and with high current demand. As regards its price, it can be found, approximately, at double the price of alkaline batteries.

Although the nickel-metal hydride batteries are optimal for a final version of the system, we acquire alkaline batteries of 9 V and 640 mAh for the set of sensors in this preliminary system version for testing reasons. As the supply voltage of those batteries is above the operative voltage of an XBee node, we may connect a filtering and voltage regulation system at 3.3 V for a correct operation of the entire circuit.

**Figure 6:**
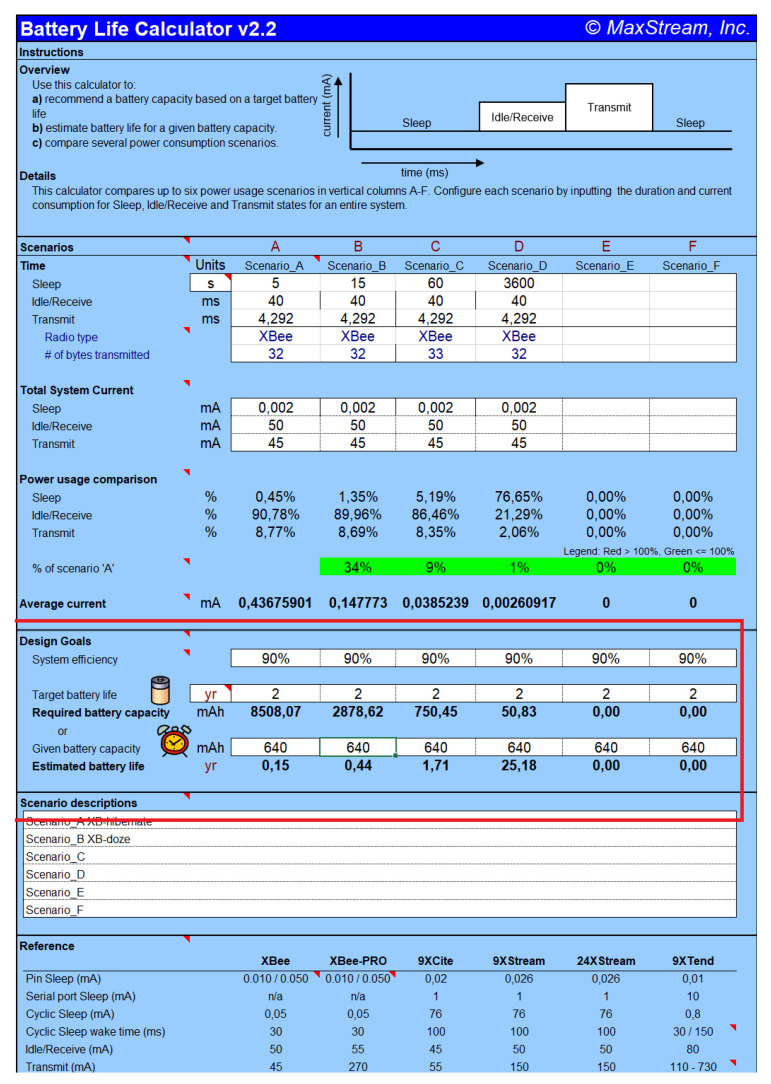
Battery consumption sheet.

An interesting advantage about using the Digi XBee node is the availability of an Excel spreadsheet [32] to calculate the battery life of their nodes according to different operative configurations. Figure 6 shows the calculation for different configurations. In this case, we modified the time which the sensor stays in sleep mode from five seconds to one-hour duration.

We can observe how the most demanding configuration, 5 s, the life of the installed battery is calculated approximately 0.15 years (55 days approximately). With 15 s, the battery life reaches 0.44 years (160 days approximately), while if the duration of this mode of low energy is one minute the battery would last almost 2 years. Finally, in long periods of time, 1 h, the battery life will last more than 25 years. Note that these calculations do not include the consumption of the rest of the electronic element (i.e., thermistors and resistors).

### 2.6. Waterproof Case

**Figure 7:**
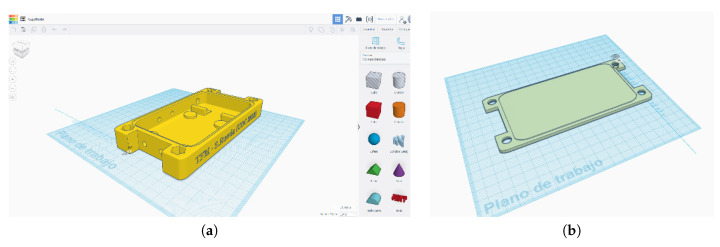
3D design of the waterproof case. (**a**) The whole case. (**b**) The cover.

Ase these nodes will be placed in a harsh industrial environment (i.e., outside the dome or near-heating systems), we decided to build a suitable waterproof case. We decided to build the waterproof case using a 3D printer. We used polylactide as material to print the waterproof case. The design was performed using the software Tinkercad. This design of the waterproof case avoids unnecessary spaces where elements may crash and save space from the whole product. Figure 7a shows the design procedure of the whole waterproof case and the Figure 7b the cover. Tinkercad allows the exportation of files in *stl* format accepted by the 3D printer (model Leapfrog BOLT PRO 3D) to generate, finally, G-code files which contain the coordinates of the waterproof case to inject the material successfully.

### 2.7. Data Collector System

As mentioned in Section 2.2, nine XBee modules are used as sensing nodes while the tenth unit will be the collector. Therefore, the collector node will be the connection bridge between the sensing nodes, through radio frequency signals, and a micro-controller board. As our micro-controller board, we used a Raspberry Pi 2 Model B because of performance, easy deployment, and it can be placed in a given location permanently.

## 3. Results

This section presents how our nodes have been prototyped, developed, and tested.

### 3.1. Prototype Implementation

**Figure 8:**
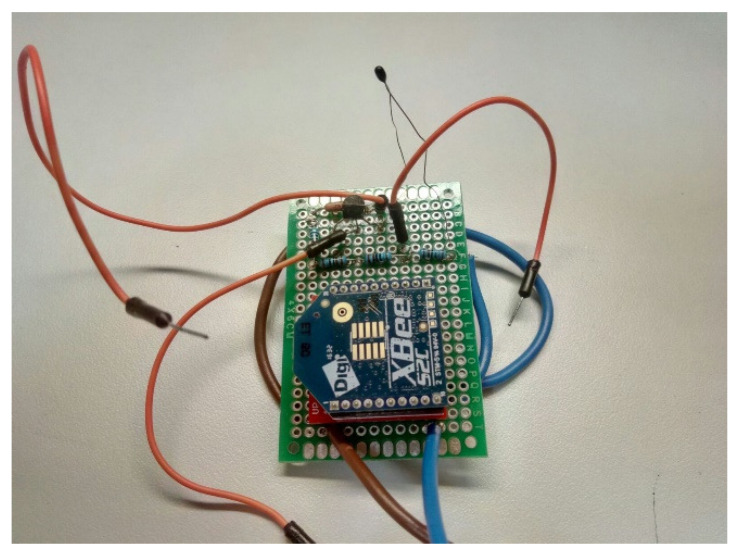
The first prototype of a sensor node made in a pre-drilled board. Three inputs were used: one with a PR103J2 sensor, one with an LMT84 sensor and another with a voltage divider to validate the values of the measurements.

The electrical diagram (cf. Figure 3) was tested in a pre-drilled prototyping PC board, before requesting the final version PCB. Figure 8 presents the test in a pre-drilled board with satisfactory results.

**Figure 9:**
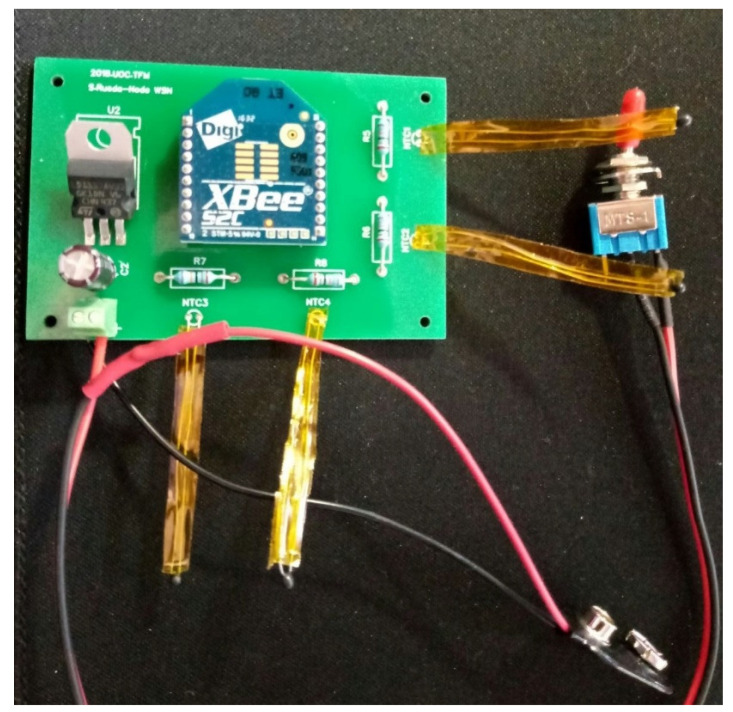
PCB with all integrated components. Thermistors are covered with Kapton tape to prevent short circuits and protect these components as they will be more exposed.

This phase of the prototyping includes a temperature sensor comparison test. We used three inputs from the board as follows: one with a PR103J2 thermistor, one with an LMT84 and another with a voltage divider to check the precision in the conversion of the analogue value. The results were fully satisfactory so it is decided to send to manufacture the PCBs. Figure 9 shows the manufactured PCBs with the communication and sensing modules.

In addition, Figure 9 presents the board with all the required elements. Note that the thermistors were covered with Kapton tape to prevent short circuits and protect the thermistors against external causes.

**Figure 10:**
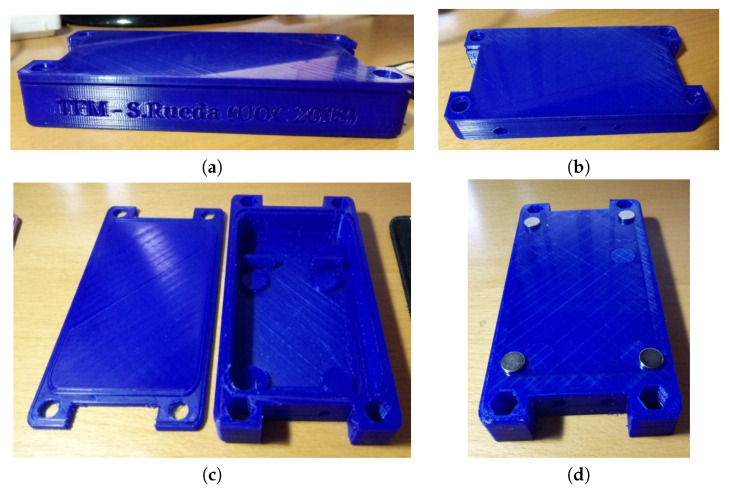
Box designed to house the electronics. (**a**) Side view of the case; (**b**) the holes for the output of two thermistors and switch are observed; (**c**) the inside of the box with the compartment to house the 9 V battery, the PCB brackets, and two other front holes for the thermistor output; (**d**) back of the case with its four neodymium magnets to fix the case to the metal dome.

In parallel, the waterproof cases were printed to house the electronic elements. Figure 10a represents the side view of the case with the cover placed on the top. Figure 10b shows a bird view of the case where we can observe two holes to accommodate appropriately the thermistors. Figure 10c presents the interior of the case with the different compartment areas to house the battery and the PCB board. Finally, Figure 10d shows the case with four neodymium magnets attached to fix the node to the metal dome. Although each neodymium magnet can hold more than 2 kg of weight, we preferred to avoid catastrophic scenarios in which a fallen may damage the expensive observatory lenses. The dimensions of the waterproof case are 155 × 80 ×20 mm built with 100% renewable materials.

**Figure 11:**
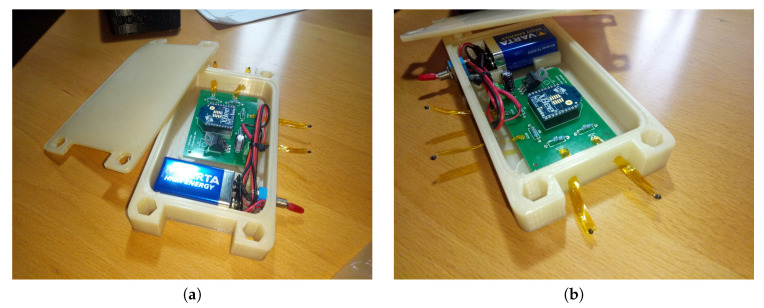
Electronics accommodated inside the waterproof case. (**a**) Battery view. (**b**) Board view.

After the construction of the waterproof case, we can accommodate all the electronic systems inside it. Figure 11 shows the completion of the node where both the battery and the PCB board fit perfectly inside the 3D-printed case. It is possible to observe how the node is completed from the battery side (a) and from the PCB side (b). The compartment for the 9 V battery prevents its movement once the box is closed, and the installation of a microswitch gives tension to the whole node. The case has been designed to be as watertight as possible, therefore, a small coupling socket between the box and the cover is incorporated, and it is possible to close it with M8 hexagonal thread screws.

**Figure 12:**
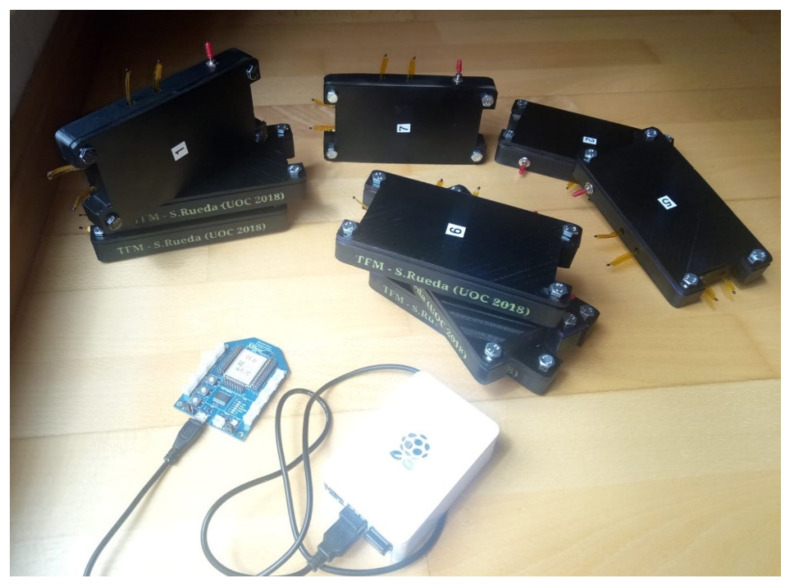
The upper part shows all nine nodes, bottom-left part the collector node, and the bottom-right case is the raspberry pi micro-controller.

We perform the same procedure of 3D-case printing and electronics integration to all nine end nodes. Figure 12 shows the whole sense acquisition system. The upper part of Figure 12 presents all nine complete sensing nodes. We decided to paint all cases in black for uniformity and avoid possible light reflections. In the bottom-left part of the Figure 12 appears the collector node connected to the raspberry pi micro-controller board directly.

### 3.2. Laboratory Tests

Once the complete system was developed, the following laboratory tests were performed.

- **Validation of the radio signal range**: The nodes were separated more than 20 m from the collector node without loss of data, which is totally satisfactory as no separation between end nodes and the collector node greater than 10 m is expected within the dome
- **Validation of the system of fixation by magnets**: The nodes were attached to metal surfaces and subjected to vibrations and traction without any node being detached. As indicated, each node is equipped with four neodymium magnets that support more than 2 kg each.
- **Precision in the measurement**: The values captured by the nodes were contrasted with a meteorological station and with an infrared thermometer. Although there were no large discrepancies in the measurements, the differences in the measurements varied, according to the acquisition system used, between 0.5 and 1 °C. This makes this test simply give us a guiding idea of the validity of the developed system. Finally, as has been said, we chose, on the one hand, to measure the input voltage, which was done with a good quality multimeter (Fluke FLUKE-87-V/EUR) in all entries comparing their value with that shown by the software developed, and on the other hand, repeating the measurements placing different sensors at the exact same point without, in any case, appreciating significant differences (at some time, at most of the order of hundredths of a degree). As has been said, in the long term it would be interesting to perform a more exhaustive calibration of the sensors; however, this point is validated, especially if even in the case that the error may exist, it is an error of the same order in all the nodes and in all the sensors and therefore it is not significant if we work with differences between them. Moreover, indicate that the nodes have been subjected to temperatures ranging from 3 °C (inside a refrigerator) to 37 °C with a correct response in the measurement.
- **Robustness of the software**: Software uses a multithreaded implementation, one thread for each node and one for failure events detection. In addition, Zigbee WSN-based networks are resilient to nodes’ failures. The proposed system has been left running with all nodes for periods of time ranging from a couple of hours to three days, and in its final version, the software is considered fully stable and ready for production.
- **Battery life with different node parameters**: The Digi XBee nodes provide few parameters to adapt their communication behavior according to the goal. Depending on those communication parameters, the battery life varies. One of the most important parameters affecting the battery life is the sleep mode. The sleep mode is used to reduce energy consumption and therefore increase the life of the batteries. They are based on the use of what is known as indirect messages, these types of messages occur when, during one of those “dream” cycles, the coordinator or router must store the message to be sent to the node when it wakes up, this is possible because when this awakening of a node occurs, it sends a message to its parent device to know if there is any data or request pending. The coordinator node is only able to save an indirect message for each node, that is, it cannot save a message queue per node. In addition, the coordinator stores the message during a period of 2.5 times the time marked in the time before sleep (ST) parameter. A remote device is activated once per period and sends a request to the coordinator to know if there is an indirect message for him. If it does not exist, the node will return to sleep mode during another new cycle, this process consumes approximately 30 ms per cycle. On the other hand, if there is a pending message for the remote node, the coordinator will transmit this direct message, and the remote node will remain awake according to the ST parameter, during which time the entire message must be received. We have performed six tests (see Table 2) modifying the following parameters to study how long the battery life stays.
  - **Sleep period (SP)**: period of time that a final device is in sleep mode (valid values from 320 ms to 28,000 ms).
  - **Number of cyclic sleep periods (SN)**: Indicates the number of cycles that the device will consider for the rest periods and therefore for the timeouts, this is done by setting the sleep options (SO) parameter that will be seen later and allows the extension of the resting periods of the nodes (valid values from 0x1-0xFFFF).
  - **Time before sleep (ST)**: Defines the period for the reception of the indirect message before entering sleep mode (valid values from 0x1-0xFFFE).
  - **Sleep options (SO):** This parameter admits to values: 2-always awake during the whole ST period and 4-Enables extended sleep mode (sleeps during SP×SN milliseconds).

We can observe in the Table 2 how doubling the sleep period (configurations 1 and 2) the battery life duration goes from 12.83 to 13.80 h, only a 7% improvement. By increasing the sleep time to 60 s (configuration 4), the battery increases by 489% more with respect to the configuration 1. If the time before sleep is reduced from 8 to 1 s and the number of cyclic sleep periods is doubled (configurations 2 and 3), the battery life is increased 85%. By tripling the number of cycles (configurations 3 and 5), the duration of the battery life increases by 282%. Finally, extending the sleep mode (SO = 4) and tripling the sleep period (configurations 3 and 6), the battery life is increased by 292%. The configuration 6 provides a battery life duration of 100 h against an almost 13 h using the configuration 1. Therefore, we choose configuration 6 because of its battery life duration. We have tested almost all possible configurations, however, we just added those more important to ease the reading flow. The differences between the results in Table 2 and those present in Section 2.5 are because of the Digi data and circuit factors.

We have observed different results extracted from our system to assure an optimal measurement precision, security, communication range, and battery life improvement. The next step is the performance of real experiments to test our system in a realistic situation (c.f. Section 3.2).

### 3.3. Real Location Experiment Design

**Figure 13:**
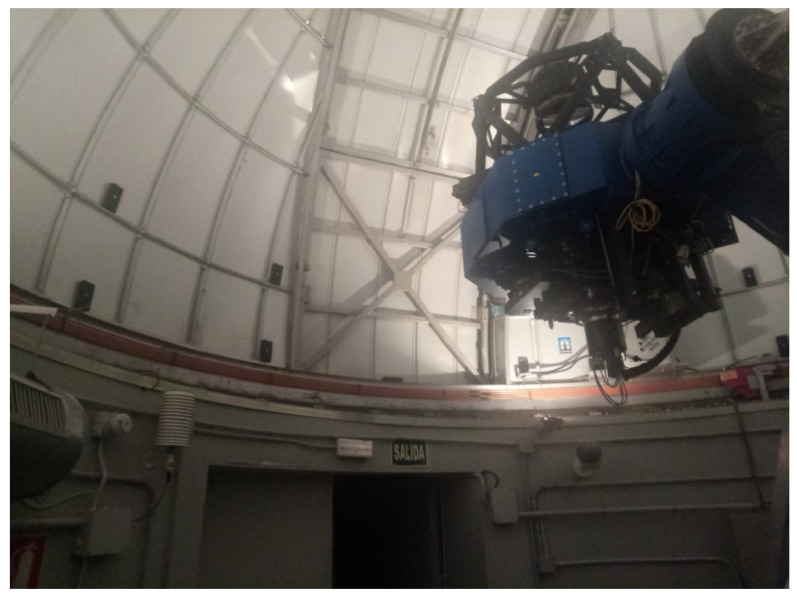
Real deployment within the D080 of all nine sensing nodes. Four nodes are observed on each side of the input slot and a node in the lower-middle part of the same.

This section presents the experiments design in the real location. In order to perform real experiments, all nine sensing nodes were distributed as shown in Figure 2, with a separation among nodes between 80 cm and 120 cm). In Figure 13, we can observe the real distribution along the dome. Two groups of four nodes are placed on each side of the input slot and the ninth node in the lower-middle part of the same.

**Figure 14:**
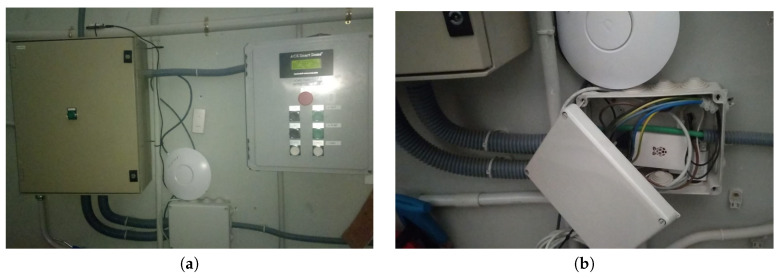
Location of both collector node and raspberry board. (**a**) General view. (**b**) Distribution panel view.

Both the collector node and the Raspberry board, as it requires a continuous feeding, are connected from the electrical grid directly. Figure 14a presents the electric grid elements and the position of the collector node. Figure 14b shows the place where the Raspberry board will be accommodated. We decided to put the Raspberry inside a distribution panel to protect it.

### 3.4. Software Monitoring System

Besides all the hardware implementation described in Section 3.1, we developed a software system to collect, process, and visualize the sensed data. We mainly used Python as a central logical unit to control all the processes. Moreover, we used the proprietary software from Digi, XCTU, to configure all node parameters. Other software tools include InfluxDB (open database), Telegraf (data collection) and Grafana (data analysis and visualization). The description of all software tools is out of the scope of this paper. However, all the codes can be found in the following GitHub profile [33].

**Figure 15:**
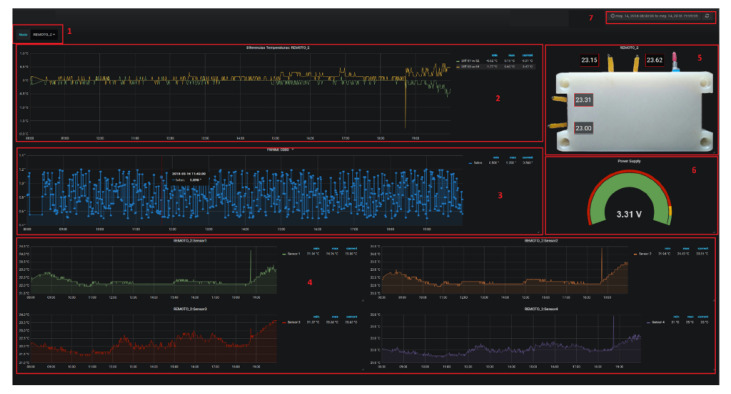
Grafana visualization example of nodes transmitted information.

As a visualization, Figure 15 presents the transmitted information by the sensing nodes, collected by the node collector and processed posterity in Python. This screenshot belongs from the Grafana tool where data can be easily interpreted for a fast behavior identification.

Our visualization design is composed of the following.

1. Drop-down menu that allows you to select a specific node, from REMOTE_1 to REMOTE_9.
2. This panel shows the temperature difference in °C between pairs of sensors of each node, that is, sensor1-sensor2 and sensor3-sensor4. On the right side, the current, maximum, and minimum values.
3. Here the FWHM values are shown in arc seconds of each image acquired by the T080 telescope, on the right, the maximum, minimum and current values are also shown. The intuition behind the FWHM measurement can be explained in the following example. Assuming an observed star through a telescope, the star can be represented by its brightness vs the pixel position. If you represent those parameters in a plot, you will get a Gaussian like curves that come up and drop back down to the background value. Statistically there are no zero values on both sides of the curve, therefore it is almost impossible to measure the true diameter of a star; therefore, what astronomers do is they look at the height of the Gaussian representation, they divide it in half and then they measured the width of the star at that 1/2 point, this line is a measure of the full-width at half of the maximum height of the star’s profile. To determine the contribution in seconds of arc that the differences in temperature in ${}^{{}^{\circ}}$C have on the FWHM, it will be necessary to carry out statistical studies that allow us to obtain correlation values between both physical parameters; however, we expect to reach some simple relationships such as those obtained by the works of D. Blanco [4].
4. In this section there are four graphs, one for each thermistor of the selected node, showing the temperature history in ${}^{{}^{\circ}}$C, the maximum, minimum, and current value.
5. An image of the selected node with the current temperature value (${}^{{}^{\circ}}$C) calculated for each sensor is displayed.
6. Current value of the node’s power supply voltage.
7. From here you can choose the viewing period, allowing us to choose a specific date, or periods of time so wide ranging from the last seconds to the last years.

**Figure 16:**
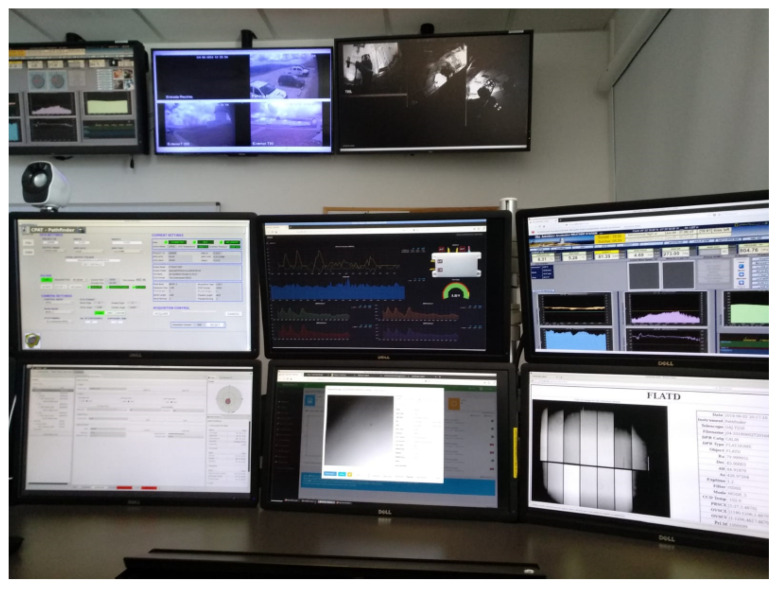
Monitoring system.

Note that the monitoring system is accessible from any control post of the observatory and also from any remote location through a virtual private network and the corresponding security certificate. Figure 16 shows the monitoring system from one of the posts in the observatory’s main control room. Currently, the observatory has three control rooms: one remote located in the headquarters of the city of Teruel, another smaller in the observatory itself, mainly for the control of the T080 telescope, and another main one, also located in the observatory, from which the staff usually control all the telescopes and systems.

### 3.5. Local Seeing Calculation

Finally, considering all available information in the monitoring system, available thanks to the developed hardware prototype, we present an average approximation of local seeing calculation using our capture temperature data from all nine nodes, for indoor temperature variations, for almost four hours. We must clarify that we performed the calculations using Equation (10) by D. Blanco [4]. Considering four PR103J2 thermistor sensors, two aimed to collect dome temperature and two aimed to collect ambient air temperature, the equation is

(10) $$Seeing=0.1\times \Delta T=0.1\times {\left(abs\left(\frac{Temp1+Temp2}{2}-\frac{Temp3+Temp4}{2}\right)\right)}^{1.2}$$

The reasoning behind Equation (10) is that each node has four thermistors. Their placement in the board is to work in pairs for acquiring ambient air and dome temperatures. Therefore, we measure the average temperature difference. Note that there are different possibilities to achieve the same goal; however, we decided this approach the most relevant.

**Figure 17:**
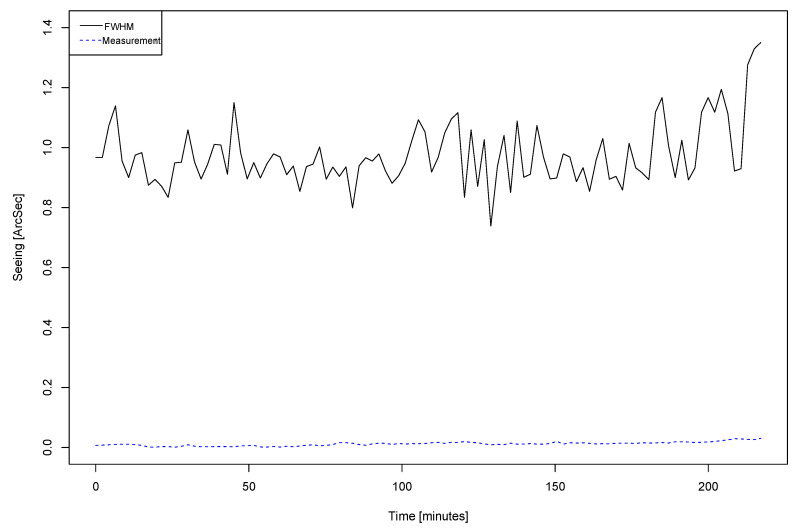
Local seeing calculation from FWHM and measurement.

Here, we assumed that the behavior of our dome is exactly the same as the Canada-France-Hawaii Telescope [4,5]. However, it will require further data capturing and analysis to refine the Equation (10). Figure 17 presents a plot displaying the seeing where the x-axis represents the time in minutes while the y-axis displays the seeing in arcsec. We compared the calculated local seeing from all distributed nodes (blue-dashed line) using the Equation 10 with the FWHM seeing (black-solid line).

We can observe how the average contribution along the four hours of measurement is 0.023 arcsec. In terms of how measurement precision can affect the results, sensor error order is 0.05 ${}^{{}^{\circ}}$C, resulting about thousandths of arcsec of the possible error, that means negligible since we usually consider tenths of arcsec.

Although it could seem a slight contribution in general terms, it is a good preliminary contribution considering the OAJ mean seeing, being able to perform more complex estimations depending on dome areas, gradients, in others. Our insight behind this procedure is to create gradient maps both inside and outside the dome for further studies.

This plot shows how much the entrance window from the dome reduces the image quality, being able to detect even slight differences with respect to the FWHM line. Thereby, we can plan tasks to improve that degradation. One possibility is to try to take into account the turbulence in the input window, and this can be normally corrected using a wind shield made with a defined permeability (in our case is 50%) minimizing the input turbulence. In addition, the best way to control the temperature of the entrance window is to establish a strategy that allows homogenizing the dome temperature.

## 4. Discussion

Along with this paper, we have described the prototyping of an open-source wireless sensing system to measure the inner temperature of a dome from astrophysical observatories and improving the local seeing. With respect to the related work (cf. Section 1), this paper is foregoing in terms of innovation using wireless sensor networks, by using them to make available the improvement of local seeing in an astrophysical observatory. Taking into account the developed work, relevant considerations can be highlighted:

- We had to test our circuit using a pre-drilled board since the breadboard returned unstable results.
- We obtained consistent temperature measurements using our system. In addition, we contrasted the results with a meteorological station and an infrared thermometer.
- We find out that both the protocol and the XBee devices chosen lack an internal and synchronized management of the sleep mode. In our configuration, each node goes into sleep mode and does not wake up until its own internal clock indicates that it should do so. Since these clocks work autonomously, there is no way within the protocol to synchronize the equipment to take data in unison. This is a problem for long periods of sleep where data can be requested from a node and it does not respond until it wakes up, while another node can be awake and respond with your data at that moment. Although these modules support activation from sleep mode by using an external pin, this would require the use of another micro-controller to perform this management, increasing complexity and consumption.
- Configuring sleep mode periods of five seconds, the shifting is not higher than three seconds, not critical in our application.

By using nine nodes and distributing them along the dome, we can create a map where turbulence effects are produced because of temperature variances. Considering that effect, we can perform the following tasks for temperature adequacy in the dome to obtain a precise local seeing. Those may be performed to achieve the following.

- Redirect the air conditioning to an inconsistent area of the dome for homogeneity.
- Rotate the dome according to the season and hours of sun to keep a constant temperature along the dome.
- Study the thermal conductivity of the dome. In case of differences between the inside and outside of the dome, isolating material may be implied.
- Study if the entrance window of the dome gap creates turbulence phenomena. In this case, we can install a fabric with 50% permeability to reduce the air intake (also known as a windshield).
- Study the thermal evolution of the dome and propose ventilation routines. Therefore, create ventilation models adapted to the climatic conditions.

Future applications of our sensing system inside the OAJ include:

1. **Study of the vertical temperature gradient**: Locate sensors every meter in height to study if there is an important thermal gradient that produces convection movements in the air. If this is the case, we would seek to homogenize the temperatures, such as, for example, installing fans that mix the air during the day (obviously, it should allow time for the atmosphere to be stable at night) or use the air conditioning of the dome (it has addressable gratings) in order to create an environment as homogeneous as possible.
2. **Study of the temperature gradient in the horizontal plane of the dome**: Locate sensors by the perimeter of the surface of the dome, ideally at the height of the instrument, to see if any part of the dome heats up much more than the other. The idea here would be to study whether heating by solar radiation or the orientation of the dome produces any considerable effect (the north side is noticeably colder than the south side). With the data obtained, we can make an estimate of whether it is necessary to move the dome during the day in order to get more homogeneous temperatures or some other action.
3. **Study of point heat sources**: As mentioned, inside the dome there are different points that generate heat, such as electrical panels, motors and lighting systems. The idea would be to place sensors in the vicinity and in the elements themselves and thus study if there is any temperature gradient, if this is so, and it is considered important, there are solutions such as cooling those systems (i.e., in the observatory there are cooling systems based on glycol water that are used to cool important heat sources).

Finally, in terms of prototype improvements, battery life is an important issue to consider. New tests for batteries have been initiated to assess the possibilities of being used in nodes. We have achieved an increase of 20% (120 h) using the Jupio JBL-9V battery and more than 900 h using the 18,650 Rechargeable 3.7 V/3500 mAh battery model.

## 5. Conclusions

Along with this paper, we present the seeing importance in astrophysical observatories to build the most complete sky map for further object discoveries. We emphasis how the local seeing provoked from the dome itself may influence the image quality. More specifically, the temperature variation is a key factor on the turbulence creation and, as a consequence, blurrier images acquisition. The literature collects many works about seeing studying in real observatories. However, so far to the best of our knowledge, no one has designed, built, and tested a wireless sensor network. This paper contributes to the astronomy community by introducing an open source, low-cost, scalable, low-consumption, and portable network to measure the temperature variation inside and outside an observatory to study the local seeing. In addition, not only we developed a hardware product; also, an open source software interface has been implemented to collect and visualize the temperature, tension and battery life from all nodes in real time, and local seeing has been calculated from our open source nodes. Finally, in order to validate new future work in terms of improving the prototype after describing experiments and results, we have performed new multiple tests to extend the node battery life, achieving an increase of 20% (120 h) using the Jupio JBL-9V battery and more than 900 h using the 18,650 Rechargeable 3.7 V/3500 mAh battery model.

## Figures and Tables

**Table 1:** Comparing performance characteristics from all tested temperature sensor.

Characteristic	TMP36	LMT84	PR103J2
Operative Voltage (V)	2.7–5.5 1.5	No limit (1)
Calibration unit	${}^{{}^{\circ}}$C	${}^{{}^{\circ}}$C	${}^{{}^{\circ}}$C
Scale factor (mV/${}^{{}^{\circ}}$C)	10 −5.5	16 (2)
Precision	±2 ${}^{{}^{\circ}}$C	±0.4 ${}^{{}^{\circ}}$C	0.05 ${}^{{}^{\circ}}$C (±0.1%, 10 KΩ at 25 ${}^{{}^{\circ}}$C)
Linearity	±0.5 ${}^{{}^{\circ}}$C	High linearity	No lineal (3)
Operative Temperature	−40 ${}^{{}^{\circ}}$C to 125 ${}^{{}^{\circ}}$C	−50 ${}^{{}^{\circ}}$C to 150 ${}^{{}^{\circ}}$C	−55 ${}^{{}^{\circ}}$C to 80 ${}^{{}^{\circ}}$C
Power consumption	50 μA (0.5 μA stand-by)	±50 μA (5.4 μA stand-by)	Power input dependent (4)

**Table 2:** Comparing different sleep mode parameters to study the battery life duration.

Configuration	ST (sec)	SO	SN	SP (sec)	Duration (h)
1	8	0	1	5	12.83
2	8	0	1	10	13.80
3	1	0	2	10	25.61
4	8	0	1	60	75.63
5	1	0	6	10	97.90
6	1	4	2	30	100.31
